# Hepatitis B Vaccine and Multiple Sclerosis: Cause or Coincidence

**DOI:** 10.7759/cureus.29941

**Published:** 2022-10-05

**Authors:** Kunjal Luhadia, Mohamed Abugrin, Roudabeh Kiani, Abolfazl Ahmady, Jaswinder Virk, Kanica Yashi

**Affiliations:** 1 Internal Medicine, Bassett Medical Center, Cooperstown, USA; 2 Cardiology, Bassett Medical Center, Cooperstown, USA

**Keywords:** hepatitis b vaccines, vaccines, engerix vaccine for hepatitis b, neurological side effects of vaccines, hepatitis b, hepatitis b vaccine adverse effects, adverse effects of vaccines, tumefactive multiple sclerosis, hepatitis b vaccine and multiple sclerosis, hepatitis b vaccine

## Abstract

Multiple sclerosis (MS) is an autoimmune disease in which the body’s immune system destroys myelin causing disruption of signals from the brain to the rest of the body. MS can be triggered by a variety of reasons. In this study, we present the case of a patient who developed neurological symptoms immediately (one day) after receiving the hepatitis B vaccine. The temporality of symptoms makes us question whether there is an association between the hepatitis B vaccine and MS. We would like to emphasize the importance of considering MS as a side effect of the hepatitis B vaccine and adding MS to the differential diagnosis of a patient who presents with neurological symptoms after receiving the hepatitis B vaccine.

## Introduction

One of the great achievements of contemporary medicine is the globalization of vaccines and mass immunizations. Current guidelines recommend routine vaccination for 17 vaccine-preventable diseases that occur in infants, children, adolescents, or adults [1], one of which is hepatitis B. Since its inception, universal hepatitis B vaccination programs have been implemented in 168 countries with outstanding efficacy and safety records [2]. However, vaccine-related disorders are inevitable, and there are some case reports of the hepatitis B vaccine causing new cases or relapses of multiple sclerosis (MS) or other demyelinating disorders such as Guillain-Barré syndrome [2]. Long-term and rare adverse events can be challenging to detect and identify as studies may lack the necessary length of follow-up or sample size to unveil them [3].

In one study titled *Vaccinations and Multiple Sclerosis*, the authors mentioned that it is possible that there is some molecular mimicry between the hepatitis B antigens and myelin proteins, or maybe a non-specific activation of the immune system that could lead to adverse neurological outcomes among these patients [4].

The patient in this case report was diagnosed with tumefactive MS. Tumefactive MS is a rare, aggressive form affecting one or two out of every 1,000 people with MS [5].

Tumefactive MS presents with a large intracranial lesion, greater than 2 cm with mass effect, perilesional edema, and/or ring enhancement with gadolinium contrast [6]. It responds well to high doses of corticosteroids [5]. Tumefactive MS can progress to relapsing-remitting MS, and when that happens the patient can be treated with disease-modifying therapies [5].

## Case presentation

A 29-year-old female presented to the emergency department with dizziness, slurred speech, and impaired coordination for six days. She reported that her symptoms began one day after receiving the hepatitis B and flu vaccine. Her condition gradually worsened with changes in mental status, abulia, and generalized weakness. Her medical history was significant for bipolar disorder not on medication, hepatitis C, and substance use disorder with a recent injection of suboxone pills.

On examination, she was afebrile with a heart rate of 55 beats/minute, blood pressure of 122/72 mmHg, respiratory rate of 13 breaths/minute, and oxygen saturation of 100%. Cardiovascular, respiratory, and abdominal examinations were unremarkable. Neurologic examination was pertinent for slurred speech, dysmetria, shuffling gait, and weakness more prominent in the right extremities. On further neurologic examination, she had intact gross sensations and cranial nerve functions, deep tendon reflexes were normal, her neck was supple, and no signs of meningeal irritation were noted.

**Figure 1:**
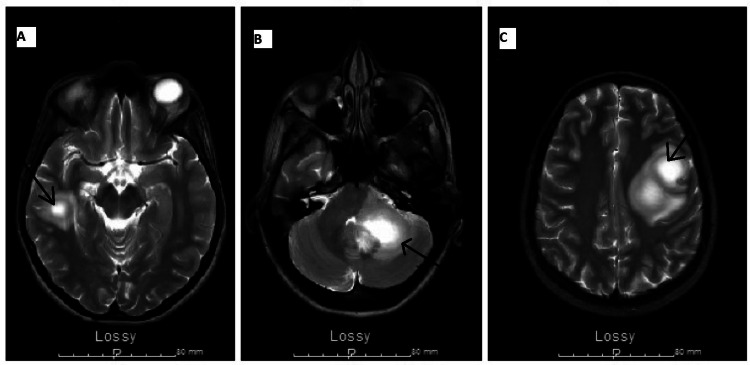
Transverse sections of T2-weighted brain magnetic resonance imaging showing enhancing lesions in the right cerebral (A), left cerebellar (B), and left cerebral hemispheres (C).

Lab results on admission were unremarkable. A computed tomography (CT) scan of the head without contrast revealed multifocal underlying space-occupying lesions. Magnetic resonance imaging (MRI) of the brain with and without contrast revealed multiple rim-enhancing lesions of varying sizes in the bilateral cerebral and left cerebellar hemispheres. The lesions showed mild surrounding edema (Figures 1A-1C).

Other imaging modalities were unremarkable, including CT scans of the chest, abdomen, pelvis, and transthoracic and transesophageal echo. The infectious workup was normal, including blood cultures, human immunodeficiency virus, syphilis serology, toxoplasma serology, and Lyme serology. Hepatitis C RNA was undetectable.

Because of the imaging results and recent intravenous drug use, the patient was started on dexamethasone, vancomycin, and meropenem for 12 days [7]. Following no improvement, she underwent a stereotactic burr hole biopsy/drainage of left frontal and left cerebellar lesions. The frozen section was significant for hypercellular glial tissue, and cultures showed no growth. Tissue histology revealed macrophages and lymphocyte infiltrates, with myelin loss and gliosis suggestive of a malignant/tumefactive form of MS. The patient was switched to methylprednisolone for seven days and then tapered on oral prednisone. Subsequently, the patient was treated with plasmapheresis; with the plasmapheresis, she saw an immediate improvement in her symptoms including being able to get out of bed and being able to feed herself. She received a total of six rounds of plasmapheresis.

However, over the course of two years, she did not develop any further neurological attacks, and her repeat brain MRI one year and two years later did not show any new interval lesion development. The patient needs to be continually monitored for any further neurological symptoms and new lesions on brain MRI for a confirmed diagnosis of MS. However, on initial presentation, it seemed like the patient had tumefactive MS.

## Discussion

MS is an autoimmune inflammatory demyelinating condition affecting the brain and spinal cord. Disease presentation can vary widely and includes dizziness, vertigo, sensory disturbance, vision problems, motor weakness, urinary dysfunction, and impaired cognition, depending on which area of the central nervous system (CNS) is affected [8]. MS has multiple subtypes; the most common is relapse-remitting MS, accounting for 87% of cases [8]. The subtype, in this case, is Marburg (tumefactive) MS, a severe progressive form of the disease first described in 1905 which is characterized by the development of tumor-like lesions in the CNS [9].

The etiology of MS is unknown, but both the environment and genetics appear to play a role in the disease. Environmental factors include smoking [10], vitamin D deficiency [11], and viral exposure. One of the mechanisms by which viruses can induce autoimmune diseases is molecular mimicry [12]. This patient developed neurological symptoms one day after receiving the hepatitis B and influenza vaccine. She has had multiple shots of the influenza vaccine in the past without any complications, but this was her first exposure to the hepatitis B vaccine.

Vaccines are one of the most effective methods of primary prevention. The introduction of the hepatitis B vaccine to the United States in 1982 has led to an almost tenfold decrease in acute hepatitis B infections [13]. The safety and efficacy of vaccines are well documented in the literature. Still, there is an established association between vaccines and neurologic demyelinating syndromes, particularly Guillain-Barré syndrome with the influenza vaccine [14] and MS with the yellow fever vaccine [15]. The hepatitis B vaccine received scrutiny due to a surge in MS cases following mass immunization in France. Since then, several studies have been conducted to assess possible correlations. While most studies demonstrated no increase [16-18] or no significant increase in risk [19-21], some did show a significant correlation and increased risk [22-24]. The proposed pathogenic mechanisms include non-specific immune activation, toxic contaminants, or molecular mimicry [4]. A study assessing amino acid similarities between hepatitis B vaccine and myelin found shared homologies between myelin basic protein, myelin oligodendrocyte glycoprotein, and hepatitis B surface antigen [25].

It is also interesting to note that the patient in this case report received the ENGERIX-B/RECOMBIVAX HB hepatitis B vaccine, and one case-control study involving children based in France reported that although the hepatitis B vaccine did not increase the risk of MS in general, “the Engerix B vaccine appeared to increase the risk, specifically for confirmed MS, in the longer term.” The study mentioned that their results required confirmation in future studies [24].

## Conclusions

The immune response in MS can be triggered by various environmental factors, one of which may be vaccines. It is critical for healthcare providers to be aware of the different side effects of vaccines and consider vaccines as the potential cause of the abnormality. In this case, the temporal relationship between receiving the hepatitis B vaccine, and the onset of symptoms makes us wonder whether there could be a causal relationship between the two. However, further studies are needed to assess the association between the hepatitis B vaccine and MS. Meanwhile, we would like to emphasize that clinicians should also suspect MS in patients presenting with neurologic symptoms days to weeks following hepatitis B vaccination.
